# Difficult weaning from cardiopulmonary bypass after surgical VSD closure: An unusual rare case

**DOI:** 10.1002/ccr3.3486

**Published:** 2020-11-06

**Authors:** Mohammad Mahdavi, Sanaz Asadian, Hossein Shahzadi, Mahdi Daliri, Nahid Rezaeian, Yaser Toloueitabar

**Affiliations:** ^1^ Rajaie Cardiovascular Medical and Research Center Iran University of Medical Sciences Tehran Iran; ^2^ Department of Radiology Rajaie Cardiovascular Medical and Research Center Iran University of Medical Sciences Tehran Iran; ^3^ Department of Congenital Cardiac Surgery Rajaie Cardiovascular Medical and Research Center Iran University of Medical Sciences Tehran Iran

**Keywords:** anomalous left coronary artery connected to the pulmonary artery (ALCAPA), cardiopulmonary bypass, cardiovascular abnormalities, ventricular septal defect

## Abstract

The connection of the left coronary artery to the pulmonary artery may be asymptomatic due to high pulmonary vascular resistance in the context of left‐to‐right shunts. Before the repair of the mentioned anomalies, coronary anatomy must be defined.

## Graphical abstract

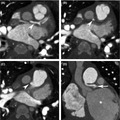

## 1 INTRODUCTION

The anomalous left coronary artery connected to the pulmonary artery (ALCAPA) may be asymptomatic due to persistently increased pulmonary vascular resistance when associated with left‐to‐right shunts. Therefore, when the correction of cardiac anomalies with severe pulmonary hypertension is planned, the preoperative determination of the coronary artery anatomy is compulsory.

ALCAPA is a congenital coronary anomaly responsible for cardiac failure due to myocardial infarction in early infancy.^1^ Because of its fatal nature, ALCAPA must be corrected immediately after diagnosis. Although frequently an isolated anomaly, it has been reported to occur in association with a variety of other congenital cardiac abnormalities such as patent ductus arteriosus (PDA), ventricular septal defect (VSD), coarctation of the aorta, and tetralogy of Fallot.^2^, ^3^, ^4^ When ALCAPA exists in association with left‐to‐right shunts, patients may be asymptomatic in infancy owing to persistently elevated pulmonary vascular resistance, which maintains coronary perfusion. Therefore, PDA ligation or VSD closure may acutely decrease pulmonary vascular resistance and prove disastrous for patients with ALCAPA.^5^ We herein present a case of VSD and PDA, complicated in the postoperative course by undiagnosed ALCAPA in the preoperative course.

## 2 CASE HISTORY

A 10‐month‐old male infant was referred to our center with the impression of a large subaortic VSD, a moderate‐sized PDA, and pulmonary hypertension. Poor feeding and failure to thrive were noted. The patient was on oral medication (digoxin, furosemide, and enalapril).

On physical examination, he was 6200 g in weight and 67 cm in height with normal blood pressure, pulse rate, and body temperature. Additionally, tachypnea was detected. On cardiac examination, a precordial holosystolic murmur was auscultated. The other body systems were normal on examination.

Transthoracic echocardiography revealed mild left atrial enlargement, mild mitral regurgitation, moderate tricuspid regurgitation (peak pressure gradient =67 mm Hg), right ventricular enlargement, tricuspid annular plane systolic excursion (TAPSE) of 10 mm, a large subaortic VSD (9 mm) with a bidirectional shunt, no evidence of aortic coarctation, and a left ventricular ejection fraction of 55%. Cardiac computed tomography angiography (CCTA) confirmed the same anatomical findings.

In surgery, the PDA was suture‐ligated and the VSD was closed via the right atrial approach with autologous pericardium in a continuous running manner. At the end of the operation, the patient was weaned from cardiopulmonary bypass in sinus rhythm but with difficulty with the aid of a high dose of inotropes.

## 3 DIFFERENTIAL DIAGNOSIS, INVESTIGATIONS, AND TREATMENT

**FIGURE 1:**
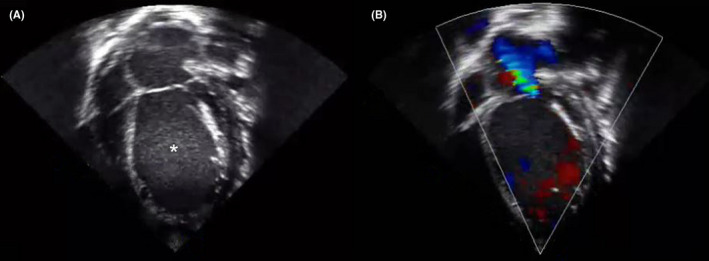
Postoperative transthoracic echocardiography. A, apical 4‐chamber view reveals left ventricular enlargement (asterisk). B, apical 4‐chamber view with doppler mode represents severe mitral regurgitation

Postoperative transthoracic echocardiography revealed severe left ventricular dysfunction, moderate mitral regurgitation, and severe pulmonary hypertension (Figure 1); however, there were no right ventricular dysfunction and residual VSD.

The patient was transferred to the pediatric intensive care unit (PICU) with a stable hemodynamic status, and his sternum was kept open. In the next 2 postoperative days, his clinical conditions and echocardiographic findings showed no change. Therefore, CCTA was performed to seek the cause of the left ventricular dysfunction, and it revealed ALCAPA: the left main coronary artery originated from the pulmonary artery adjacent to the left sinus of Valsalva.

## 4 OUTCOME

The patient was transferred to the operating room, where the translocation of the anomalous artery to the aorta was carried out. Thereafter, he was placed on extracorporeal membrane oxygenation.

In the next 10 days of the PICU course, the patient's left ventricular function improved but unfortunately, he developed clinical and laboratory signs of multi‐organ failure secondary to sepsis and eventually expired.

## 5 DISCUSSION

Anomalous left coronary artery connected to the pulmonary artery, also known as Bland‐White‐Garland syndrome, is a rare congenital disorder with an occurrence rate of 1 in 300 000 live births. It is the leading cause of myocardial infarction in children, with death rates reaching 90% in early childhood if it remains untreated. Thanks to extensive intercoronary artery collaterals, however, 10%‐15% of cases can survive to adulthood.^6^

The after‐birth decline in pulmonary vascular resistance leads to reduced myocardial perfusion. In ALCAPA, the collaterals develop gradually between the right and the left coronary arterial systems, resulting in coronary steal. Aortic oxygenated blood passes in the right coronary artery, avoids the myocardium's high‐resistant vessels, and runs into the low‐resistant pulmonary artery at the end.^7^ Nonetheless, less coronary steal and sufficient coronary perfusion to the myocardium exist in the presence of increased pulmonary artery pressure in conditions such as PDA or VSD. Left ventricular dysfunction may develop postoperatively if a coronary artery with an anomalous origin goes undiagnosed prior to the related lesion repair, resulting in major morbidity and mortality, which is similar to the situation that we encountered.^4^, ^8^

Blood in the pulmonary artery and, consequently, the left coronary artery in patients with ALCAPA with a concomitant left‐to‐right shunt is more oxygenated than that in isolated ALCAPA. Therefore, myocardial ischemia does not occur until the time of left‐to‐right shunt corrective surgery, which explains the occasional catastrophic postoperative courses of these cases described in the literature.^2^, ^4^, ^8^

The association between ALCAPA and other cardiac anomalies is sporadic and can, thus, be missed before surgery. The associated anomalies may lead to certain hemodynamics obscuring ALCAPA manifestations. As a result, myocardial ischemia appears just after the defect reparation. Moreover, in patients with undiagnosed ALCAPA, improper cardioplegic solution administration leads to an even more worsened postoperative left ventricular function.

In the presented patient, as was described above, an immediate reduction in pulmonary artery pressure and, accordingly, a decrease in the perfusion of the left main coronary artery occurred after PDA and VSD closures, leading to massive left ventricular myocardial infarction and cardiac failure after the weaning from cardiopulmonary bypass.

**FIGURE 2:**
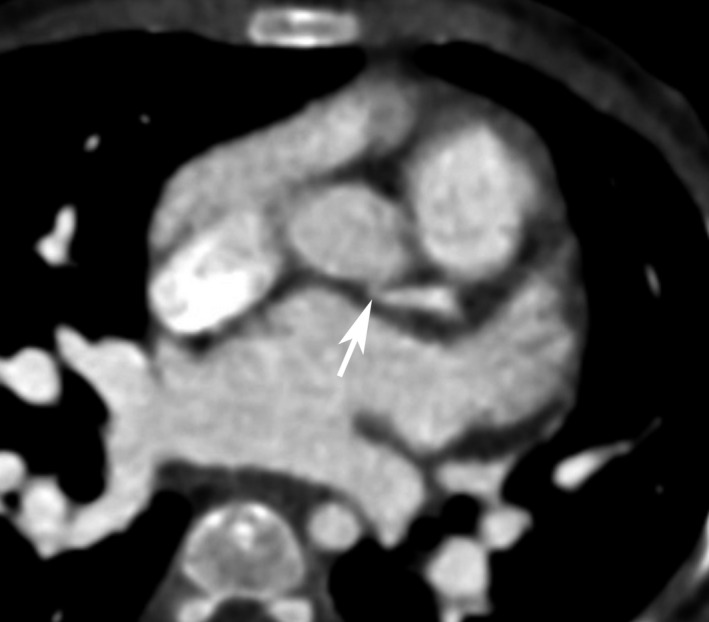
Nonelectrocardiogram‐gated preoperative coronary computed tomography angiography shows the site of close contact of the proximal part of the left main coronary artery with left sinus of Valsalva (arrow) caused misinterpretation

**FIGURE 3:**
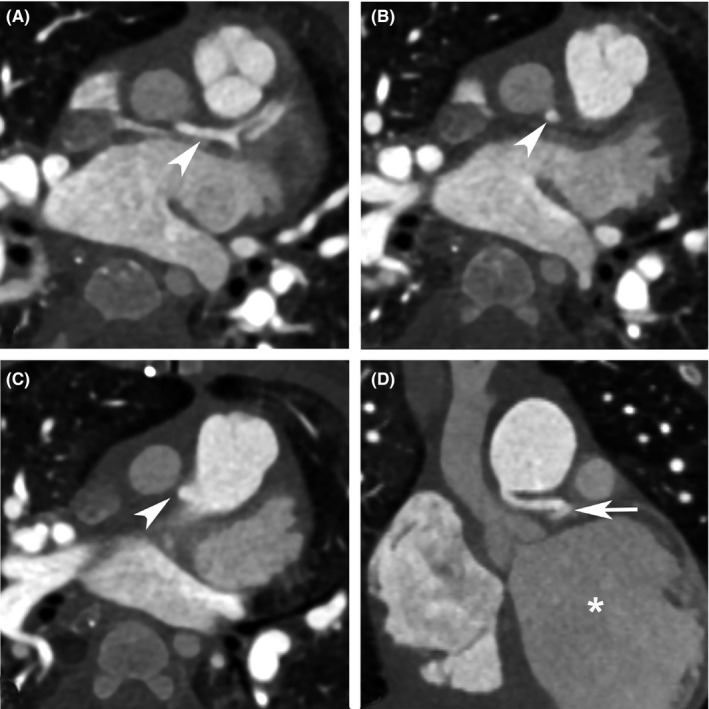
Electrocardiogram‐gated postoperative coronary computed tomography angiography reveals the course of the left main coronary artery. A, B, and C, Angulated course of the left main coronary artery (arrowheads) in three consecutive axial sections. D, Entire course of the left main coronary artery in coronal plane (arrow); note dilated left ventricle (asterisk) representing left ventricular dysfunction

In our case, the angulated and interarterial course of the proximal part of the left main coronary artery, as well as the respiratory motion artefactual effect of tachypnea in the first preoperative CCTA, precluded the diagnosis of ALCAPA (Figure 2). The other probable cause for this misinterpretation was the nonelectrocardiogram‐gated protocol of the mentioned CCTA. Nevertheless, in the second postoperative electrocardiogram‐gated CCTA, precise anatomical delineation was achieved (Figure 3), which underscores the significance of careful preoperative anatomical and functional evaluations using echocardiography and electrocardiogram‐gated CCTA before congenital cardiac operations. As was mentioned by Lee et al, prospective electrocardiogram‐gated CCTA is the modality of choice for pediatric cardiac evaluation from both quality and radiation dose standpoints.^9^

In conclusion, when the correction of congenital cardiac anomalies with severe pulmonary hypertension is planned, a precise determination of the coronary artery anatomy in preoperative evaluation is mandatory.

## CONFLICT OF INTEREST

The authors declare no conflict of interest.

## AUTHOR CONTRIBUTIONS

YT and MM: contributed to acquisition of data, drafting the manuscript, final revision of the manuscript, and participated sufficiently in the work. SA, HS, MD, and NR: contributed to acquisition of data, drafting the manuscript, and participated sufficiently in the work.

## ETHICAL APPROVAL

The report was performed in accordance with the ethical standards as laid down in the 1964 Declaration of Helsinki and its later amendments or comparable ethical standards. Also, it is in accordance with institutional policies in this subject. Informed Consent: Written informed consent has been obtained from the patient (parents).

## Data Availability

The datasets generated during the current report are available from the corresponding author on reasonable request.
